# Assessment of Gel-Based Thermochromic Glazing for Energy Efficiency in Architectural Applications

**DOI:** 10.3390/ma17164047

**Published:** 2024-08-14

**Authors:** Kai Zeng, Chang Xue, Jinbo Wu, Weijia Wen

**Affiliations:** 1 Materials Genome Institute, Shanghai University, Shanghai 200444, China; jinbowu@t.shu.edu.cn; 2 Faculty of Materials Science, Shenzhen MSU-BIT University, Shenzhen 518115, China; 3 Department of Physics, The Hong Kong University of Science and Technology, Clear Water Bay, Kowloon, Hong Kong

**Keywords:** thermochromic glazing, architectural energy conservation, intelligent materials, energy efficiency, environmental sustainability

## Abstract

With the increasing global focus on energy efficiency and environmental sustainability, intelligent building materials such as thermochromic glazing have emerged as a hot topic of research. The intent of this paper is to explore the utilization of gel-type thermochromic glazing within the realm of architectural energy conservation calculations. It conducts an exhaustive examination of the material’s attributes, its capacity for energy savings, and the obstacles encountered in real-world applications. Through simulation studies and case analyses, this paper assesses the energy efficiency of gel-type thermochromic glazing across various climates and suggests strategies for optimization. The study revealed that the incorporation of gel-based thermochromic glazing leads to a marked reduction in energy usage within buildings, an improvement in indoor comfort levels, and significant environmental advantages.

## 1. Introduction

Amidst the relentless increase in global energy demand, the construction industry stands out as one of the most substantial consumers of energy [1,2]. Statistics reveal that the energy expended on buildings accounts for almost 40% of the entire world’s energy usage [3], a proportion that has persistently increased with the rapid pace of urbanization. The lion’s share of a building’s energy footprint is allocated to systems for heating, air conditioning, illumination, and the utilization of electrical devices [4]. As a result, the imperative to augment the energy performance of buildings and curtail energy usage has emerged as a critical issue on the global stage [5,6].

Thermochromic glazing is an intelligent glass that automatically adjusts its transmittance in response to changes in ambient temperature [7]. This type of glass typically incorporates tiny thermochromic materials [8], such as vanadium oxide, hydrogels, or solid-state PVB materials. The optical properties of these materials change as the temperature increases, reducing the transmittance and thereby lowering the indoor temperature; conversely, when the temperature decreases, the transmittance increases, increasing the indoor lighting and warmth. This automatic modulation capability allows thermochromic glazing to naturally regulate the indoor environment without the need for external energy sources.

The application potential of thermochromic glazing is immense [9], particularly in the realm of energy conservation. By diminishing reliance on artificial lighting and air conditioning systems, thermochromic glazing can significantly curtail a building’s energy consumption. Moreover, it can increase the comfort level of indoor environments by naturally regulating light and temperature, resulting in more pleasant living and working conditions. In sustainable architectural design, thermochromic glazing is regarded as a crucial innovative technology that is expected to have widespread application in future constructions. However, thermochromic glazing with VO_2_ as the tuning material does not result in pronounced energy-saving effects. In contrast, gel-based thermochromic glazing, with its adjustable transition temperature and superior energy-saving parameters postobscuration, is the focus of this paper because of its application and evaluation in building energy conservation calculations.

Exploring the developmental strides in thermochromic glazing involves a concentrated examination of the material’s characteristics, its mechanisms for energy conservation, its implementation in architectural case studies, advancements in current technologies, and the prospective trajectories of upcoming research endeavors. The light permeability of materials at the heart of thermochromic technology is adjusted in accordance with their temperature-responsive properties. Notably, vanadium oxide (VO_2_) stands out as a highly studied material [10,11], with its phase transition occurring at temperatures nearly identical to those commonly found in indoor environments, thereby rendering it exceptionally viable for practical architectural engagements. On the frontier of material innovation, the exploration of alternative substances such as tungsten-infused vanadium oxide (W-doped VO_2_) reveals the capacity to fine-tune the phase transition temperature through doping techniques, thereby aligning the material’s performance with the diverse demands of various climatic contexts. In their seminal work, Kai Sun [12] reported that, through adept material engineering, key parameters can be intricately tuned to enhance system performance. Yanfeng Gao [13] offered an insightful overview of VO_2_ intelligent glass, underscoring its formidable potential for contributing to the energy efficiency of architectural structures. Yamei Li [14] evaluated VO_2_ thermochromic materials featuring diverse core-shell architectures and revealed that nanotechnological refinements can markedly increase their efficacy. M. Nazari [15] illuminated how doping strategies can adjust the optical bandgap and thermal robustness of VO_2_. The increase in synthesis techniques, exemplified by the sol-gel methodology and magnetron sputtering, paves the way for the creation of thermochromic VO_2_ films with exquisitely refined temperature responsiveness.

Investigations into the energy conservation dynamics of thermochromic glazing have been conducted with an integrated methodology, merging theoretical discourse with empirical substantiation. M. Saeli and colleagues, in their analytical exploration [16], illustrated that, when juxtaposed with standard glass, the integration of VO_2_ in thermochromic glazing could effectuate a reduction of up to 10% in cooling necessities. Similarly, empirical studies have shown that the deployment of VO_2_-embedded thermochromic glazing could alleviate the dependence on artificial illumination, consequently fostering additional energy preservation. The latent capacity for energy conservation within VO_2_-embedded thermochromic glazing has captured the interest of the scholarly research domain. By tuning its light transmittance, such glazing can mitigate the reliance on cooling systems during the summer months and on heating systems in the winter. Umberto Berardi and team, leveraging the EnergyPlus simulation platform [17], delineated the efficacy of thermochromic coatings on the energy expenditure of a commercial edifice situated in the heart of Toronto, Ontario, under a spectrum of climatic conditions. These coatings were determined to attenuate the need for cooling by 8.9% while circumscribing wintertime energy deficits to a mere 1.7%. Within the realm of environmental evaluation, studies akin to those performed by S. A. Sharif [18] reveal the pivotal role that a building’s thermal dynamics—both losses and gains—play in dictating energy consumption patterns and the quality of the indoor environment. The triad of heating, ventilation, and air conditioning, coupled with lighting, consumes a substantial portion of office buildings’ total energy, with percentages reaching 33% and 25%, respectively. Despite the relatively elevated energy use and emissions associated with the manufacturing process of building envelopes, the long-term energy conservation achieved post-renovation effectively compensates for these preliminary environmental expenditures. In a forward-thinking development, Uzair [19] introduced a sophisticated multicriteria decision-making (MCDM) framework. This framework serves as a decision-support tool for consumers to identify the most advantageous balance among energy efficiency, cost reduction, and the quality of the indoor environment. It encompasses not only the simulation of energy use in sustainable construction, but also an assessment of their economic prospects, projecting that enhancements to the building fabric can yield considerable energy and financial savings over time. The investigative work of D. Aelenei [20] presents the concept of adaptive building envelopes that, through the dynamic interaction of materials, components, and systems with both indoor and outdoor stimuli, can significantly bolster a structure’s energy performance and fiscal prudence. Researchers, including Loonen [21], have proposed the concept of climate adaptive building shells (CABS) as an auspicious contender in the quest for enhanced sustainability within architectural realms. Despite the nascent stage of material systems, CABS presents a vista of untapped potential for energy efficiency and the amelioration of indoor environmental standards. This is achieved by harnessing a synergistic blend of active and passive architectural techniques within the building’s protective shell, surpassing the limitations of conventional facades. Marina [22] introduced Thermochromic Smart Glass (TSG) into a laboratory setting and conducted year-long surveillance of energy usage. The findings were conclusive, with the TSG demonstrating a marked reduction in the energy required for air conditioning and illumination when juxtaposed with standard glass alternatives. Echoing this innovation, Mohammad Salamati and associates [23] advanced the discourse with the proposition of intelligent, highly adaptive solar-saving apparatuses. These devices are capable of real-time adjustments in response to fluctuating environmental parameters and energy requirements, thereby optimizing the efficacy of indoor cooling systems to their fullest extent. Furthermore, Niima Es-sakali [24] delved into the intricacies of VO_2_ film thermochromic glazing, employing a comprehensive numerical model fortified by energy valuation and radiation analysis tools. The dynamic modulation of transparent building envelopes at structural surfaces has been shown to increase the edifice’s energy proficiency while safeguarding the well-being and comfort of its inhabitants.

Despite the considerable progress achieved in ongoing research endeavors, many challenges and avenues for future exploration persist. Central to these efforts is the refinement of thermochromic material properties, which focus on bolstering their resilience and consistency, as well as on strategies to decrease production costs, thereby encouraging wider adoption across the market. Additionally, the seamless amalgamation of these materials with intelligent building systems represents a pivotal area for future scholarly pursuits. This encompasses the exploration of methods to harmonize thermochromic materials with cutting-edge building management frameworks and an array of energy conservation technologies, all in the quest to optimize energy stewardship and increase the caliber of habitation comfort.

## 2. Methodology

### 2.1. Experimental Equipment

The experimental apparatus of our study is situated in the city of Chongqing, China, nestled within the geographical coordinates of 29°46′ North latitude and 106°39′ East longitude, an area renowned for its distinct climate that swelters in summer and chills in winter. In accordance with the protocol outlined in ASHRAE 140-2023 [25], which establishes a standard method for assessing the efficacy of building energy analysis software, our experimental setup takes the form of a sunroom. The construction of the room periphery, excluding the glass elements, incorporates prevalent materials that are in compliance with stringent energy conservation standards. These materials include but are not limited to polystyrene and acrylic insulation materials, which are recognized for their efficiency in terms of thermal retention and reduction in energy dissipation. Our study employs a standardized testing chamber integrated with an avant-garde environmental surveillance system that facilitates the exact measurement of a range of indoor climatic variables, including temperature, humidity, luminosity, and air flow velocity. The chamber is also equipped with an ensemble of sensors and logging devices engineered to acquire comprehensive data pertaining to the characteristics of thermochromic glazing. We utilized a Lambda 1050 spectrophotometer (PerkinElmer, Waltham, MA, USA) to measure the spectral characteristics of the gel-based thermochromic material in various states across the range of 300–2500 nm, and we employed a Hikvision infrared thermal imager to test the temperature imaging of the skylights. Moreover, all of the instruments have undergone rigorous calibration to ensure their accuracy.

### 2.2. Meteorological and Climatic Profile

**Figure 1:**
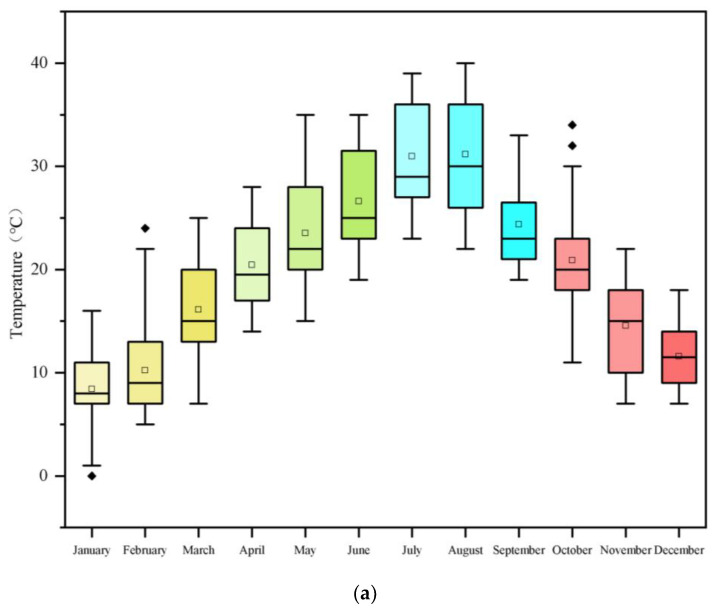

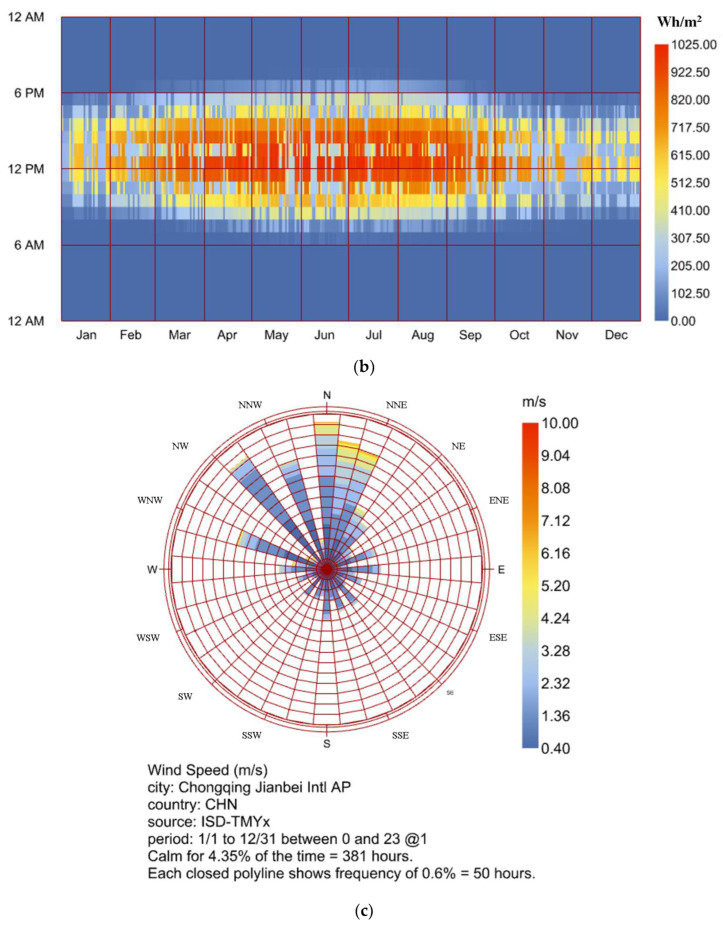
Climate characteristics of the case study model location, Chongqing City: (**a**) box plot of monthly variations in outdoor dry bulb air temperature (The special symbols are temperatures that exceed the 99.3% confidence interval of the normal distribution and are considered normal temperatures), (**b**) annual direct solar radiation diagram based on Chongqing’s solar azimuth angles, (**c**) wind rose diagram of Chongqing.

The municipality of Chongqing, nestled within the heart of China, exemplifies the quintessential climate of scorching summers and biting winters. The extrinsic meteorological data were procured from the meteorological station in the vicinity of the edifice under study. The box-and-whisker plot in Figure 1a delineates the monthly fluctuations in the ambient dry bulb temperature throughout Chongqing. In conjunction, the polar rose diagram in Figure 1b,c provides a visual depiction of the city’s annual direct solar irradiance and wind atmospheric datasets, forming the cornerstone of the boundary conditions for our numerical simulations and adeptly steering the incorporation of thermal and solar radiation parameters into the architectural model.

### 2.3. Numerical Model Description

The building energy model for the subject of our research was created via the EnergyPlus simulation engine.

We selected gel-based thermochromic glazing, whose manufacturer was Chongqing Hewei Technology Co., Ltd. (Chongqing, China), standard double-glazed units, and low-E glazed units for simulation, with output indicators including annual total energy consumption, energy savings, and daylight illuminance levels. This enabled a comprehensive comparative analysis of the energy efficiency of the building under various glass configurations, encompassing both thermal and visual comfort. Economic and environmental analyses were subsequently conducted to determine the optimal glazing conditions for hot-summer and cold-winter climates, as well as the return on investment.

In the proposed numerical model developed with EnergyPlus, the conduction transfer function (CTF) method is employed to simulate heat transfer through building components. The CTF method uses a simplified analytical model to calculate the heat transfer process, eliminating the need for mesh refinement typically associated with computational fluid dynamics simulations. By leveraging the CTF method, our numerical model ensures computational efficiency while providing accurate predictions of building energy performance.

In the simulation using EnergyPlus (24.1.0), the parameters are as depicted in Table 1 and Table 2.

### 2.4. Static and Dynamic Glass Characteristics

#### 2.4.1. Implementation of Standard Glass Installation

The thermal properties of the standard glass types used in this study, such as the U value, solar heat gain coefficient (SHGC), and visible transmittance (VTL), are derived from the most widely used glasses in the local Chongqing market. These standard glasses include double-glazed units with air cavities and low-E double-glazed units with air cavities.

#### 2.4.2. Implementation of Gel Thermochromic Glass

**Figure 2:**
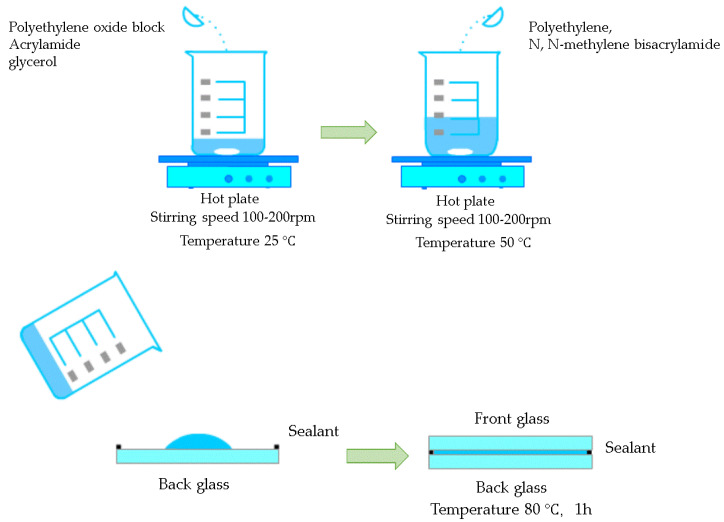
Fabrication process of gel-based thermochromic photochromic glass.

**Figure 3:**
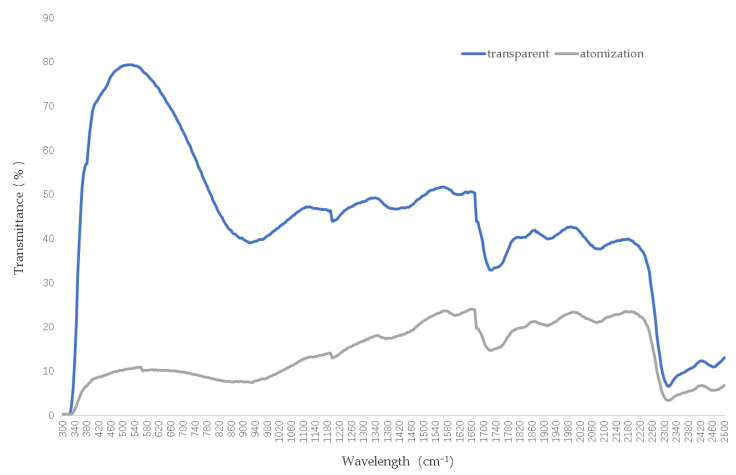
Transmission curves of gel-based thermochromic glazing in clear and obscured states.

The specific preparation method for gel-based thermochromic glass is as follows: polyoxyethylene block copolymers, acrylamide, glycerol, polyethylene, and N,N’-methylene bisacrylamide were accurately weighed and stirred at 100–200 rpm in deionized water. The mixture was poured into the glass cavity and heated to 80 °C for 1 h to form a gel, as shown in Figure 2. A thermochromic gel layer was added to the outer layer of the glass, with a microcavity used to inject the thermochromic gel into the cavity, thereby forming a double-glazed unit with an air cavity. The thermal and optical properties of the gel-based thermochromic double-glazed windows used in this study are illustrated in Figure 3. The U value of the gel-based thermochromic double-glazed glass is 2.4 W/(m^2^·K), with an SHGC of 0.70 in the clear state and 0.18 in the obscured state.

**Figure 4:**
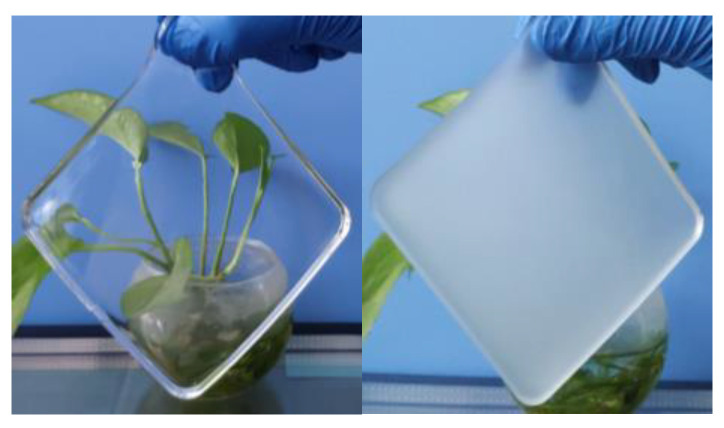
Comparison of the transparent and obscured states of the gel-based thermochromic material.

Compared with VO_2_-based thermochromic materials, gel-based thermochromic materials exhibit significant variations in visible light transmittance and the solar heat gain coefficient between their transparent and obscured states. This can be observed from the spectral curves in the figure, which depict the two states in Figure 4. When gel-based thermochromic materials reach a certain temperature, the thermochromic gel becomes completely obscured, turning milky white. Upon cooling to a specific temperature, it reverts back to a transparent state.

### 2.5. Thermal Comfort Assessment

EnergyPlus simulations were used to determine the thermal environment for standard winter and summer conditions. Actual tests were conducted to measure the environmental temperatures on typical meteorological days in summer and winter, complementing the thermal comfort assessment.

### 2.6. Visual Comfort Assessment

Visual comfort was assessed through illuminance maps and daylight glare sensors, with the sensors placed at a height of 0.8 m in the thermally active zones of the building, controlled by continuous light. The illuminance set point was set at 500 lux. The simulated illuminance levels were directly extracted from the EnergyPlus simulation engine.

### 2.7. Economic Assessment

Economic assessment is crucial for understanding the return on investment. This analysis will provide the initial investment, energy savings, maintenance costs, and expected payback period for gel-based thermochromic glazing, as well as a comparison of economic benefits with those of conventional glass and low-E glass.

Initial investment cost (C_0_): The installation cost of gel-based thermochromic glazing.

Annual energy savings (E_t_): Energy cost savings due to the high insulating performance of gel-based thermochromic glazing.

Maintenance costs (M_t_): The annual maintenance expenses for gel-based thermochromic glazing.

Energy price growth rate (g): The annual increase in energy prices.

Depreciation life (n): Service life of gel-based thermochromic glazing.

Discount rate (r): The interest rate reflects the time value of money.

The model formulas are as follows:

(1) Payback period, PP: $\mathrm{PP}=\frac{C_{0}}{E-M}$, where C_0_ represents the initial investment cost, E signifies the annual energy savings, and M denotes the maintenance costs.
(2) Net present value (NPV): $\mathrm{NPV}=-C_{0}+\sum_{t=1}^{n}\frac{E_{t}-M_{t}}{{\left(1+r\right)}^{t}}+\frac{R}{{\left(1+r\right)}^{n}}$, Et refers to the energy savings in year t, Mt represents the maintenance costs for year t, r is the discount rate, n denotes the useful lifespan, and R signifies the residual value.
(3) Internal rate of return (IRR): $0=-C_{0}+\sum_{t=1}^{n}\frac{E_{t}-M_{t}}{1+{\mathrm{IRR}}^{t}-C_{0}}+\frac{R}{{\left(1+\mathrm{IRR}\right)}^{n}}$ the IRR is iteratively calculated until the NPV approaches zero.

## 3. Results and Discussion

To assess the energy-saving potential of gel-based thermochromic glazing under various climatic conditions, we conducted a comparative analysis across several aspects, including thermal comfort and temperature regulation, light control and visual comfort, integrated environmental control, and investment payback.

In our quest to evaluate the energy-conserving capabilities of gel-based thermochromic glass across diverse climatic scenarios, a multifaceted comparative analysis was meticulously executed, encompassing dimensions of thermal comfort alongside temperature moderation, luminosity control coupled with visual serenity, cohesive environmental regulation, and investment payback.

### 3.1. Thermal Comfort and Temperature Moderation

**Figure 5:**
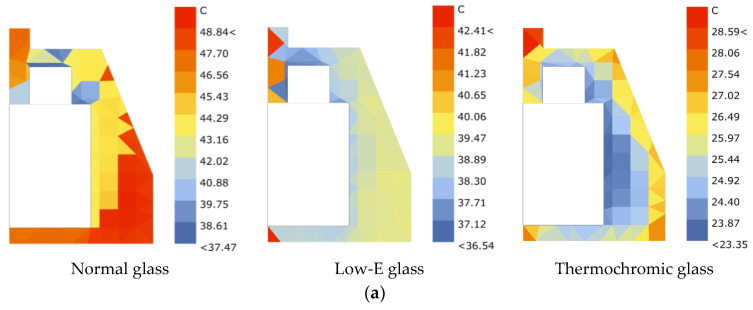

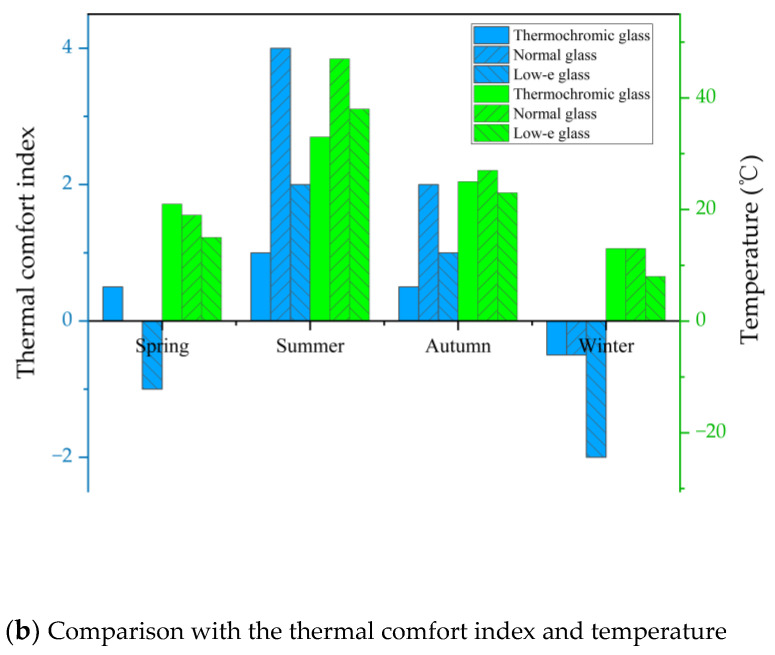
(**a**) A simulated thermal radiation table for ordinary double-glazed glass, low-glazed glass, and our focal gel-based thermochromic contender during the hot summer months. (**b**) A comparative overview of thermal conviviality and simulated climatic temperatures for the triad of glasses across the annual cycle.

The thermochromic glass in question, forged from gel matrices, champions the cause of indoor temperature equilibrium through the vigilant regulation of thermal transfer. As mercury soars, this glass acts as a sentinel, curtailing the ingress of thermal energy, and during the chill of winter, it becomes a conduit, inviting warmth into the abode. Such autonomous modulation functions as a boon, diminishing the dependency on artificial climate control mechanisms and, in turn, elevating the echelons of indoor thermal coziness. As shown in Figure 5, the thermal amicability within the confines of this glass’s embrace surpasses that of its contemporaries, with simulated indoor climes reflecting a pronounced amelioration: specifically, a marked 5-degree Celsius respite in the sweltering summer and a 3-degree Celsius advantage during the winter chill when juxtaposed with low-emissivity glass.

**Figure 6:**
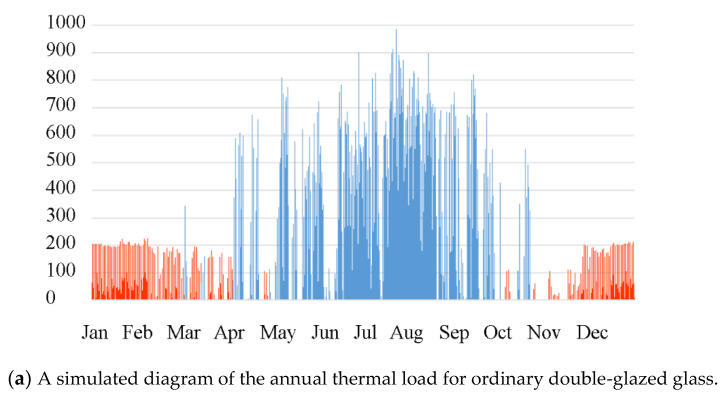

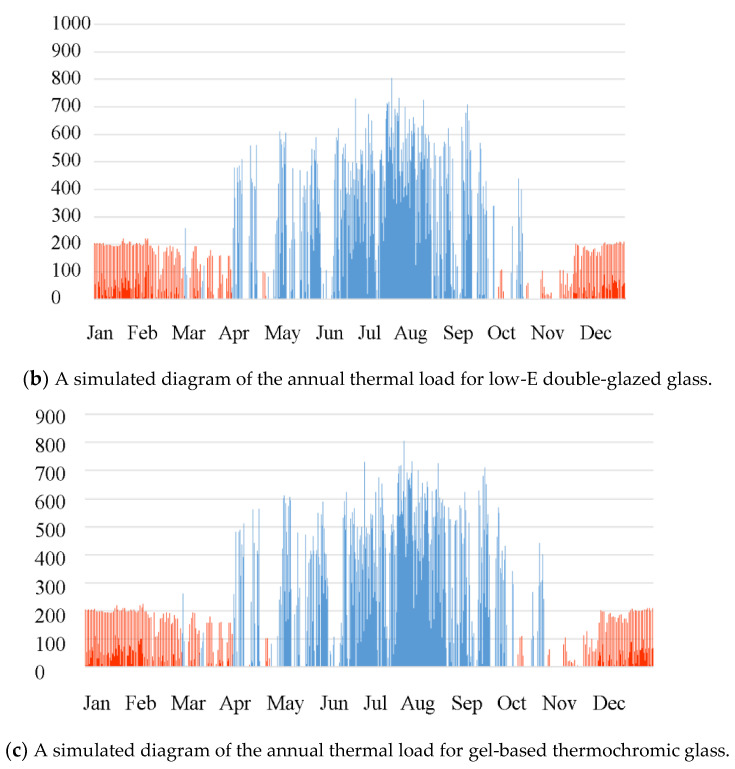
A comparative visual narrative of the annual energy expenditure for the trio of glasses. In energy consumption simulation, the blue line generally represents cooling energy consumption, and the red line represents heating energy consumption.

Figure 6 compares the annual building energy consumption for three different types of glass. The comparison shows that in the summer, the gel-based thermochromic glass is in the obscured state, with an SHGC value of 0.18. Compared with regular glass, it can reduce the air conditioning energy consumption by approximately 20%, and compared with low-E glass, it can reduce the cooling energy consumption by 12%. In the winter, the thermochromic glass is in the transparent state, performing similarly to regular double-glazed glass. In this scenario, thermochromic glass also has an advantage in terms of thermal insulation over low-E glass. This approach can effectively reduce the loss of indoor heat, thereby reducing heating energy consumption. According to simulation data, the use of thermochromic glass can reduce winter heating energy consumption by approximately 15%.

### 3.2. Illumination Control and Visual Serenity

**Figure 7:**
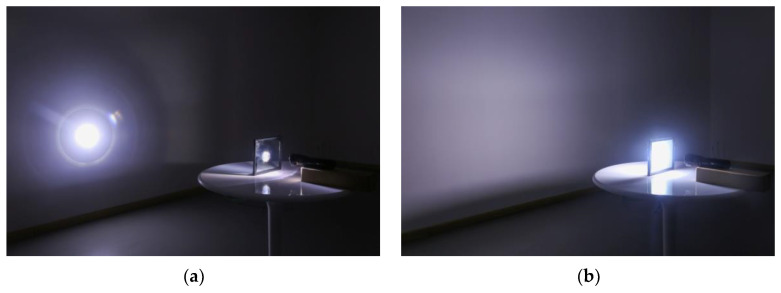
(**a**) The conditions of light incidence in the transparent state for ordinary double-glazed glass, low-E glass, and gel-based thermochromic glass. (**b**) Illustration of the conditions of light incidence through thermochromic gel-based smart glass in its frosted state.

In the meantime, while both gel-based thermochromic and low-E glasses are adept at attenuating the intensity of light, thermochromic glass stands out as a luminous guardian, significantly diminishing glare and, in turn, enhancing visual comfort, as elucidated in Figure 7. In the winter season, the thermochromic glass, which is in its transparent state, uses a bounty of sunlight, thereby increasing luminosity and visual comfort. In stark contrast, low-E glass, which is shackled by its inherent low transmittance, leads to dampened luminosity and a decrease in overall comfort. On the whole, the thermochromic glass provides a steadier visual comfort and a more fitted luminosity across the seasons, outperforming the low-E glass, which, despite its commendable energy conservation, falters in the realms of light control and comfort during the extreme seasons.

**Figure 8:**
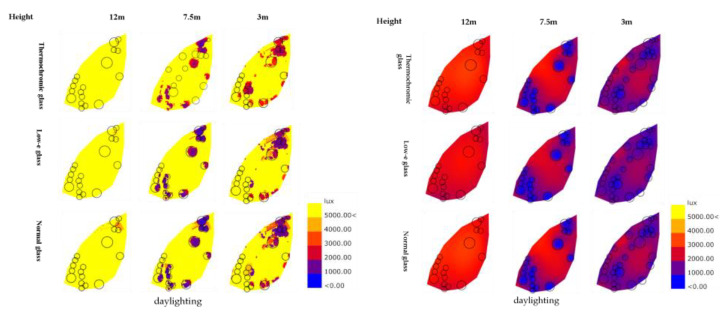
A depiction of light simulation with three distinct glass types during quintessential meteorological days of summer and winter.

As depicted in Figure 8, during the summer season, both gel-based thermochromic glass and low-E glass exhibit excellent light regulation capabilities. They significantly reduce the intensity of light entering the interior, effectively mitigating the issue of excessive brightness caused by direct sunlight. The gel-based thermochromic glass, when obscured, effectively diminishes glare, enhancing visual comfort within the space and allowing for a softer and more pleasant lighting experience. However, even in its fully misted state in summer, gel-based thermochromic glass maintains a markedly superior indoor lighting effect compared with that of low-E glass, with illuminance levels capable of reaching 5000 lux, virtually indistinguishable from the levels provided by normal and low-E glass, eschewing any significant dimming. In contrast, as winter approaches, the transparency of the thermochromic glass increases, allowing more natural sunlight to penetrate the interior. Conversely, low-E glass, with its inherently lower light transmittance, fails to effectively introduce sunlight in winter, leading to a decrease in overall light intensity and comfort. The deployment of thermochromic glass stands to substantially elevate the ambient natural light indoors, with potential illuminance spikes up to 1000 lux, sufficiently satisfying the indoor lighting criteria, whereas the low-E glass falls short in this regard, mandating additional lighting sources.

### 3.3. Integrated Environmental Regulation

Within the architectural environmental regulation paradigm, the selection of glass profoundly influences the internal climate, including temperature, luminosity, and energy expenditure. The gel-based thermochromic glazing, normal glass, and low-E glass each bring a unique suite of optical and thermal attributes to the table, all of which are pivotal to the orchestration of an integrated environmental control system.

**Figure 9:**
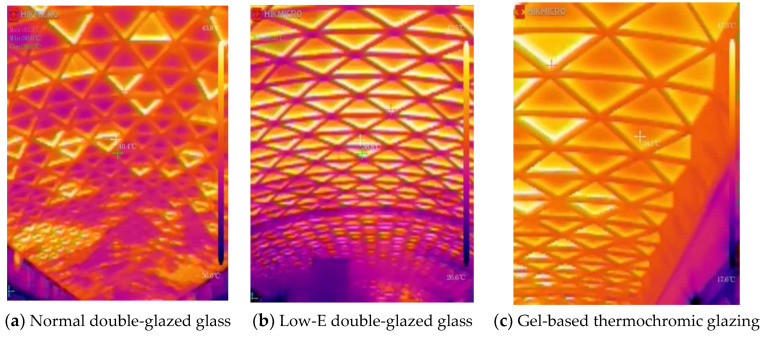
A factual representation of the internal surface temperatures of various glasses on a quintessential summer day.

As shown in Figure 9, we conducted a comparative analysis of actual tests in three commercial complexes. The surface temperature of the thermochromic glass on a typical summer meteorological day was only 38.1 °C, whereas it was 38.8 °C for the low-E glass and 40.4 °C for the regular double-glazed glass, indicating that the thermochromic glass has a significantly lower interior temperature. This demonstrates that thermochromic glass has a clear advantage in terms of reducing the indoor temperature.

**Figure 10:**
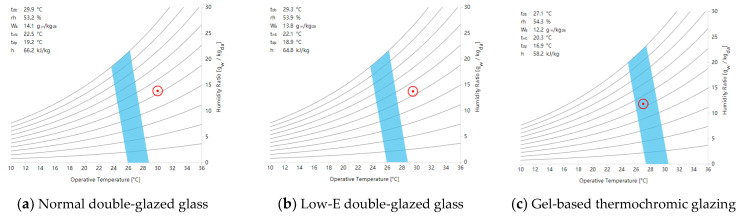
A comprehensive simulation of the environmental interaction with different types of glass within an air-conditioned setting.

Figure 10 presents a comprehensive environmental performance simulation of the three types of glass in three commercial complexes. In terms of temperature control, the indoor temperature of the thermochromic glass was 27.1 °C, that of the low-E glass was 29.3 °C, and that of the regular double-glazed glass was 29.9 °C. The commercial atrium with thermochromic glass had ample natural light, no glare, and a gentle breeze, facilitating a comfortable sensory experience. Its enthalpy difference (Δh) between the outdoor state point or mixed point and the dew point was 58.2 kJ/kg, requiring a relatively small cooling load and, thus, a smaller air supply volume. The commercial atrium with low-E glass had sufficient natural light and no glare, but the indoor air was stagnant and windless, resulting in a stuffy sensation. Its Δh was 64.8 kJ/kg, requiring a moderate cooling load and, therefore, a relatively larger air supply volume. The commercial atrium with regular glass had radiative heat, causing a stuffy sensation, with a Δh of 66.2 kJ/kg, requiring a relatively large cooling load and, thus, a larger air supply volume.

**Figure 11:**
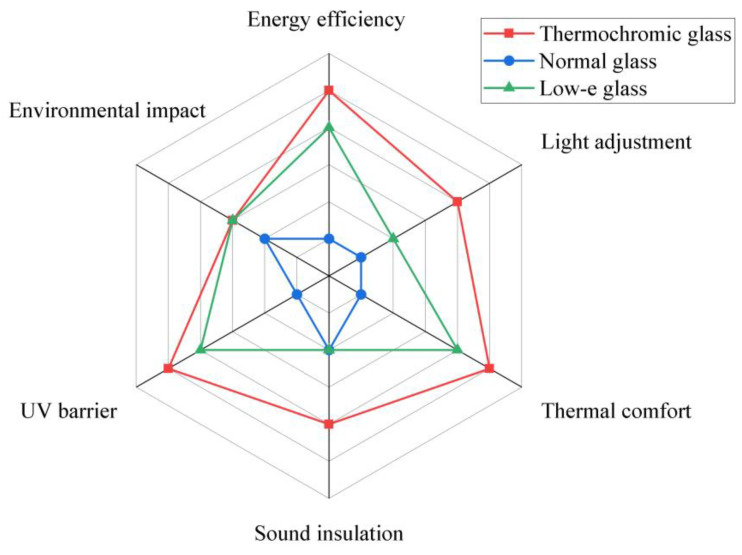
A comparative assessment of the overall environmental control efficacy of different glasses.

To conduct a comprehensive evaluation of the gel-based thermochromic glass in comparison with regular double-glazed glass and low-E double-glazed glass, particularly in terms of integrated environmental control, we assessed the following six aspects, as shown in Figure 11: energy efficiency (a metric quantifying the heat insulation and heat preservation capabilities of various glass types, with direct implications for energy usage), light modulation (the capacity of glass to regulate the permeation of light and mitigate glare), thermal comfort (the influence of glass on indoor temperature stability and its contribution to overall comfort), acoustic insulation (the effectiveness of glass in mitigating external noise interference), UV protection (the glass’s ability to shield against ultraviolet rays, safeguarding indoor items from light degradation and reducing potential radiation harm), and environmental consideration (a holistic view of the environmental impact across the life cycle of the glass, from production through disposal).

The gel-based thermochromic glass shines across the spectrum of performance metrics, particularly in terms of energy efficiency, thermal comfort, and UV protection, thereby asserting its vanguard status in the realm of comprehensive environmental control. Low-E glass, with its commendable energy efficiency and adept light modulation, presents itself as a balanced alternative, ideally suited for scenarios that prioritize high energy conservation and controlled lighting. Normal glass, regrettably, exhibits the poorest performance across all metrics, denoting its limited efficacy in environmental control; however, it may persist as a viable contender in projects where budget constraints take precedence.

For encapsulation, gel-based thermochromic glass has emerged as a tool for energy conservation and comfort, given its self-regulating capacity for light transmittance and ability to manage indoor climatic conditions across a variety of weather patterns, thus curtailing energy expenditure. While low-E glass boasts commendable energy-saving attributes, it encounters limitations in terms of light transmittance and natural light provision. Normal glass, constrained by its static nature, finds its utility in modern architecture to be circumscribed.

### 3.4. Investment Payback Estimation

In the endeavor to formulate a theoretical financial model for the investment payback period and overarching economic merits of a 1000-square-meter gel-based thermochromic glazing system for a daylighting roof, it becomes imperative to establish pertinent economic parameters. Under the assumption that the cost of gel-based thermochromic glazing is CNY 800 per square meter, complemented by an installation fee of CNY 100 per square meter, the total expenditure per square meter is CNY 900. The additional cost, compared with conventional glass, is CNY 400 per square meter, culminating in an initial outlay (C_0_) of CNY 400,000.

The annual energy conservation cost (Es) is calculated on the basis of a 10–20% reduction in air conditioning energy consumption, with a conservative estimate of 15% savings, translating to an annual expense of CNY 100,000. Given the passive nature of gel-based thermochromic glazing, the annual maintenance cost (M) is null. The energy prices are projected to increase annually by 3%, and the service life (n) of the glazing is estimated to be 25 years. The market discount rate (r) is presumed to be 5%, with a residual value (R) of the gel-based thermochromic glazing system postulated at CNY 100,000.

The payback period is demarcated at 4 years, calculated as:

$$\mathrm{PP}=\frac{C_{0}}{E-M}=4\;\mathrm{years}$$

Moreover, the net present value (NPV) is affirmative, denoted by the expression:

$$\mathrm{NPV}=-C_{0}+\sum_{t=1}^{n}\frac{E_{t}-M_{t}}{1+r^{t}}+\frac{R}{{\left(1+r\right)}^{n}}\approx 1,350,000>0$$

which is approximately CNY 1,350,000.

The internal rate of return (IRR) is approximately 12.5%, which is derived from solving the equation:

$$0=-C_{0}+\sum_{t=1}^{n}\frac{E_{t}-M_{t}}{1+{\mathrm{IRR}}^{t}-C_{0}}+\frac{R}{1+{\mathrm{IRR}}^{n}},\;\mathrm{IRR}\;\approx \;12.5\%$$

This theoretical financial model shows that the gel-based thermochromic glazing system, under the postulated assumption, promises a brief payback period coupled with a favorable net present value. These findings underscore that gel-based thermochromic glazing is not only technologically distinguished for its insulative properties, but also the most economically viable option.

## 4. Conclusions

The analysis presented here has shed light on the substantial energy conservation capabilities of gel-based thermochromic glass across a spectrum of climates.

(1) Within regions that experience sweltering summers and chilly winters, this innovative glazing material can achieve a notable reduction in energy usage by curtailing air conditioning reliance, offering year-round energy savings and potentially decreasing summer cooling demands by 20% and winter heating needs by 15%.
(2) Gel-based thermochromic glass has been proven to excel in numerous performance metrics, especially concerning energy efficiency, thermal comfort, and UV protection, thereby solidifying its vanguard status in the realm of holistic environmental control.
(3) The energy-saving potential of this glazing is contingent upon an interplay of factors, such as climatic conditions, architectural designs, the intrinsic properties of the glass, and a thorough economic assessment. Fine-tuning these elements can significantly enhance the material’s role in energy-efficient building practices.
(4) Investment return analysis reveals a swift payback period of merely 4 years for gel-based thermochromic glass, coupled with a favorable NPV and an IRR near 12.5%, indicating a compelling financial return.

Additionally, given the ability of gel-based thermochromic glass to have variable obscuration temperatures, future studies could delve into the energy conservation traits of dynamic glass at a range of temperatures and determine the most effective obscuration temperature settings tailored for diverse geographical locations. This is particularly important for structures where the ratio of transparent enclosures is substantial.

## Figures and Tables

**Table 1:** Performance parameters of various materials in the simulation software.

Parameters		Benchmark Range for Building Performance Simulation	Values Used in This Study
U value (W/m^2^ K)	Wall	≤0.35	0.35
	Roof	≤0.25	0.25
	Floor	≤0.25	0.25
	Window	≤2.2	1.8
Heating temperature setpoint (°C)		20–23	20
Cooling temperature setpoint (°C)		23–26	26
Illuminance over a task area (lux)		300–500	300

**Table 2:** Comparative analysis of the visible light and solar characteristics of three glass window systems.

Glazing System		Solar Transmittance and Reflectance			Visible Light Transmittance and Reflectance			U Value	SHGC
	State	τ(λ)	ρ_0_	ρ_i_	τ	ρ_0_	ρ_i_	W/(m^2^ K)	
Gel-based thermochromic glass	transparent	0.469	0.251	0.266	0.670	0.234	0.239	2.4	0.7
	obscured	0.057	0.351	0.430	0.073	0.421	0.477		0.18
Normal glass		0.638	0.221	0.198	0.815	0.131	0.131	2.7	0.54
Low-E glass		0.553	0.325	0.275	0.694	0.262	0.250	2.0	0.75

## Data Availability

The original contributions presented in the study are included in the article, further inquiries can be directed to the corresponding author.
